# The Effect of Intraabdominal Visceral and Subcutaneous Adipose Volume and Muscle Volume on Lumbar Vertebrae Degeneration

**DOI:** 10.7759/cureus.35940

**Published:** 2023-03-09

**Authors:** Rabia Mihriban Kilinc, Fatih İlker Can

**Affiliations:** 1 Radiology, Muğla Sıtkı Koçman University, Muğla, TUR; 2 Orthopedics and Traumatology, Muğla Training and Research Hospital, Muğla, TUR

**Keywords:** abdominal fat volume, low back pain, computed tomography, musculoskeletal disorders, lumbar vertebrae degeneration

## Abstract

Objectives: This study aimed to investigate the effect of the volume of subcutaneous, visceral, and total adipose tissue, and paravertebral muscles in patients with lumbar vertebrae degeneration (LVD) through computerized tomography (CT) images.

Materials and methods: One forty-six patients with a complaint of lower back pain (LBP) between January 2019 and December 2021 were included in the study. CT scans of all patients were analyzed retrospectively for abdominal visceral, subcutaneous, and total fat volume, and also paraspinal muscle volume measurements and analysis of lumbar vertebrae degeneration (LVD) using designated software. In CT images, each intervertebral disc space was evaluated in terms of the presence of osteophytes, loss of disc height, sclerosis in the end plates, and spinal stenosis to investigate the presence of degeneration. Each level was scored according to the presence of findings, with 1 point for each finding. The total score at all levels (L1-S1) was calculated for each patient.

Results: An association was observed between the loss of intervertebral disc height and the amount of visceral, subcutaneous, and total fat volume at all lumbar levels (p˂0.05). The amount of all fat volume measurements also showed association with osteophyte formation (p˂0.05). An association was found between sclerosis and the amount of all fat volume at all lumbar levels (p˂0.05). It was observed that spinal stenosis at the lumbar levels was not associated with the amount of fat (total, visceral, subcutaneous) at any level (p˃0.05). No association was found between the amount of adipose and muscle volumes and vertebral pathologies at any level (p˃0.05).

Conclusion: The abdominal visceral, subcutaneous, and total fat volumes are associated with lumbar vertebral degeneration and loss of disc height. Paraspinal muscle volume does not show an association with vertebral degenerative pathologies.

## Introduction

Obesity is a worldwide problem regarding its increased risk for cardiovascular diseases, stroke, diabetes, cancer, asthma, and metabolic syndrome. It also causes psychosocial disorders, decreased productivity, and economic healthcare burden [1].

Obesity has been recently admitted as a risk factor for lower back pain (LBP) which decreases physical functions, compromises the quality of life, and causes psychological distress [2]. Therefore, the etiology of vertebral disc degeneration is clinically significant. Body mass index (BMI) has been blamed for vertebral disc degeneration among both adolescents and adults [3].

Lumbar vertebrae degeneration (LVD) is a prolonged process of deterioration involving genetically determined and mechanically triggered biological factors [4]. The proceeding phase of the degenerative process is segmental dysfunction and primarily shows impairment in facet joint functions. Although aging is considered to be the only significant contributor to the process, some factors such as inflammation may have a predisposing effect on LVD [5]. As a result of the degeneration, pain, inflammation, and hypomobility originating from the facet joints begin, and the movement segment is restricted [6]. Inflammation may not only emerge as a restriction but also both pain and hypomobility altogether. Hence, cells or tissues with increasing or emerging inflammation have been investigated as a potential risk factor for LVD [7]. In some community-based general studies, higher rates of back pain and disability were detected in individuals with more fat mass, whereas those with higher lean tissue volume had no association with back pain intensity [8]. Moreover, the increased adipose volume has been shown to be associated with the risk of type 2 Modic changes in the spine resulting in back pain, which tends to have a lean mass-protective effect [9].

The relationship between fat mass in the lumbar region and intervertebral disc diseases has been reported in the literature before, but the mechanism remains unclear [10]. Not only BMI but also excessive abdominal fat mass has been associated with lumbar pathologies. There is limited information in the literature about the relationship between subcutaneous and visceral abdominal fat distribution and lumbar vertebrae pathologies [10].

Detection of adipose tissue volume and adiposity varies according to the possibilities of the researchers and the conditions provided. Methods such as densitometry, MRI, and CT are costly although they can present clear results about body fat [11]. In a variety of studies, all these measurement methods were used for the analysis of adipose tissue [2,12,13]. CT shows this complex region's bone anatomy very well and is accepted as one of the best radiological techniques for adipose tissue volume calculations [14]. Since the muscle mass is highest at L3 and L4 levels, the region that is frequently preferred in these measurements is the L3-L4 region [15]. It has also been shown in the literature that visceral fat tissue measured on a single-slice CT scan at the L4 level is significantly associated with total abdominal visceral fat volume [16].

The effects of abdominal fat tissue volume on the spinal canal and vertebrae are still unknown and a comprehensive study on this subject has not been observed in the literature. In the current study, we investigated the effect of subcutaneous adipose tissue volume, visceral adipose tissue volume, and paravertebral muscle mass on LVD through CT images of the L1-S1 vertebral levels.

## Materials and methods

Following the institutional review board approval for the study (number: 119/2019; Muğla Sıtkı Koçman University Ethical Committee), a retrospective cohort analysis was performed using the medical records of patients. For the current study, patient consent is not required. All procedures executed involving human participants were in accordance with the ethical standards of the institutional ethical committee and with the 1964 Helsinki declaration.

A total of 146 patients who applied to the neurosurgery outpatient clinic with a recent abdominal CT (max three months) because of a lower back pain complaint were included in the study. Patients with a previous history of surgery or a vertebral fracture were excluded. After excluded patients, a total of 146 patients were included in the study, of whom 90 were female (61.6%) and 56 were male (38.4%). The mean age of the patients was 51.42±13.91 (20-82) years.

**Figure 1:**
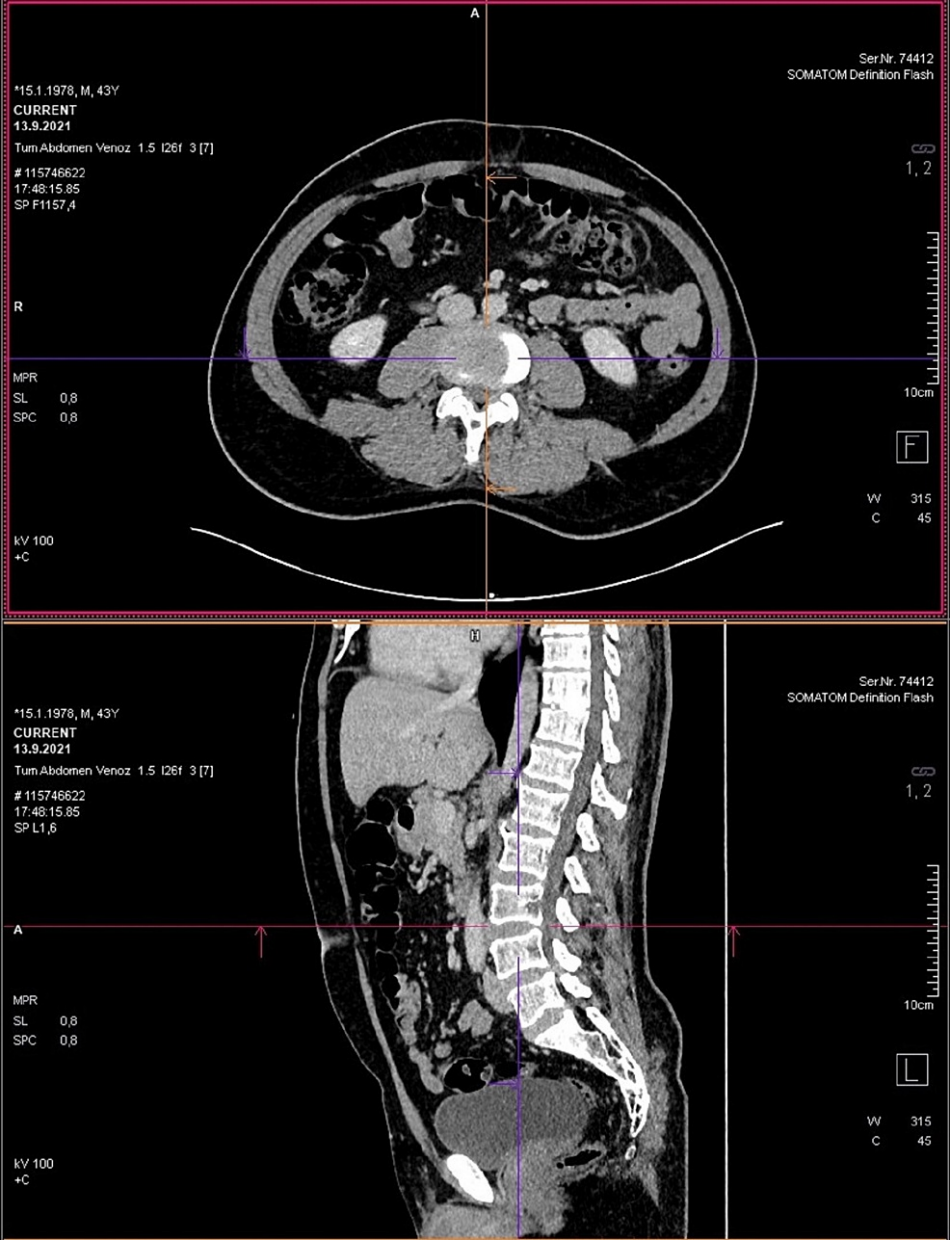
Determination of axial image at L3-L4 level and detection of region growing area on sagittal reformat images.

Lumbar vertebra CT scans of all patients were reviewed retrospectively. CT images at the level from L3-L4 intervertebral disc were analyzed for body composition of fat tissue and muscle mass volume through the dedicated CT software (Syngo.via, SOMATOM Definition Flash: Siemens Healthcare, Forchheim, Germany). The L3-L4 level was selected in sagittal reformat CT images with the software (Figure 1).

**Figure 2:**
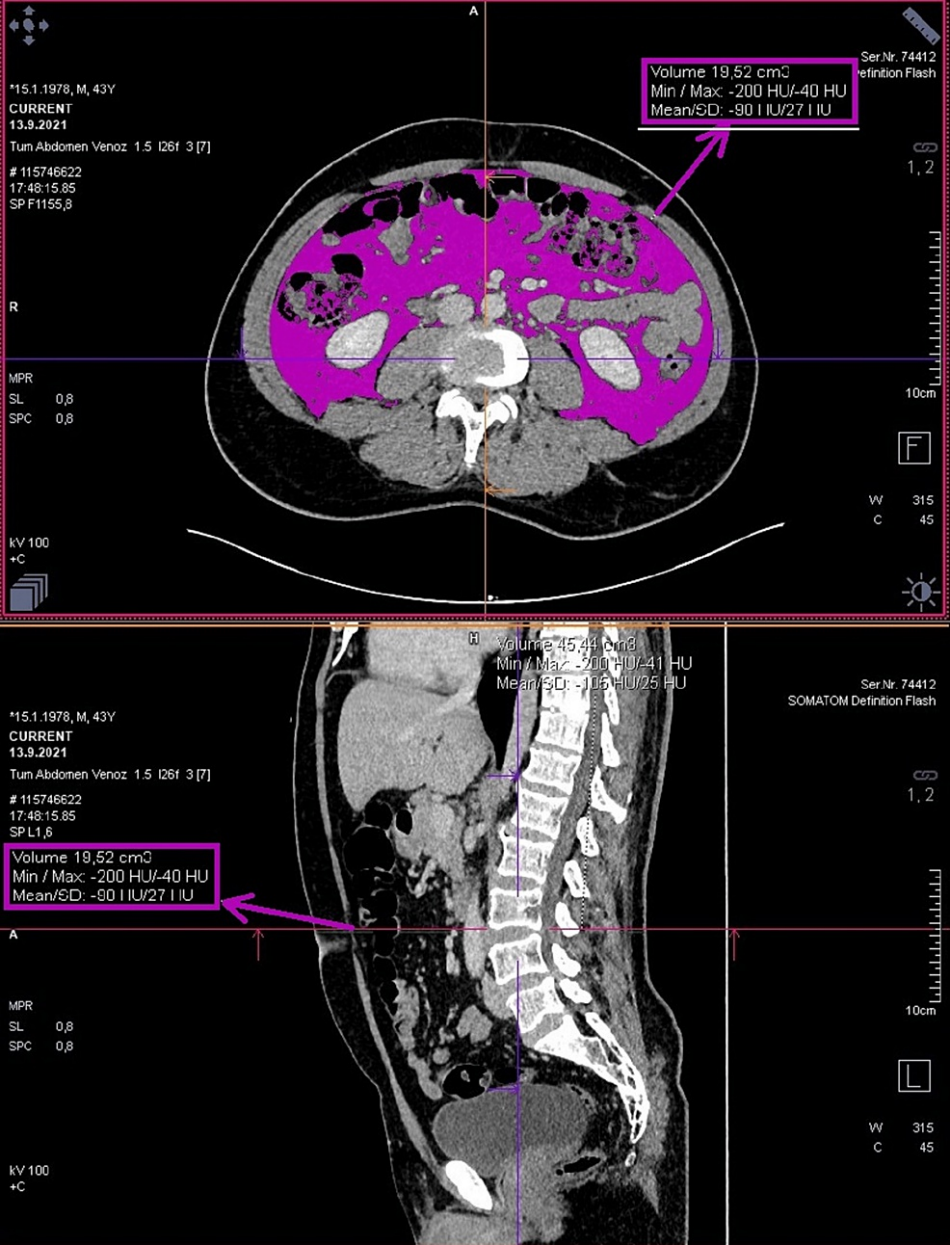
Drawing the intraabdominal area and calculating the fat volume along the inner surface of the abdominal wall, excluding the muscle planes.

**Figure 3:**
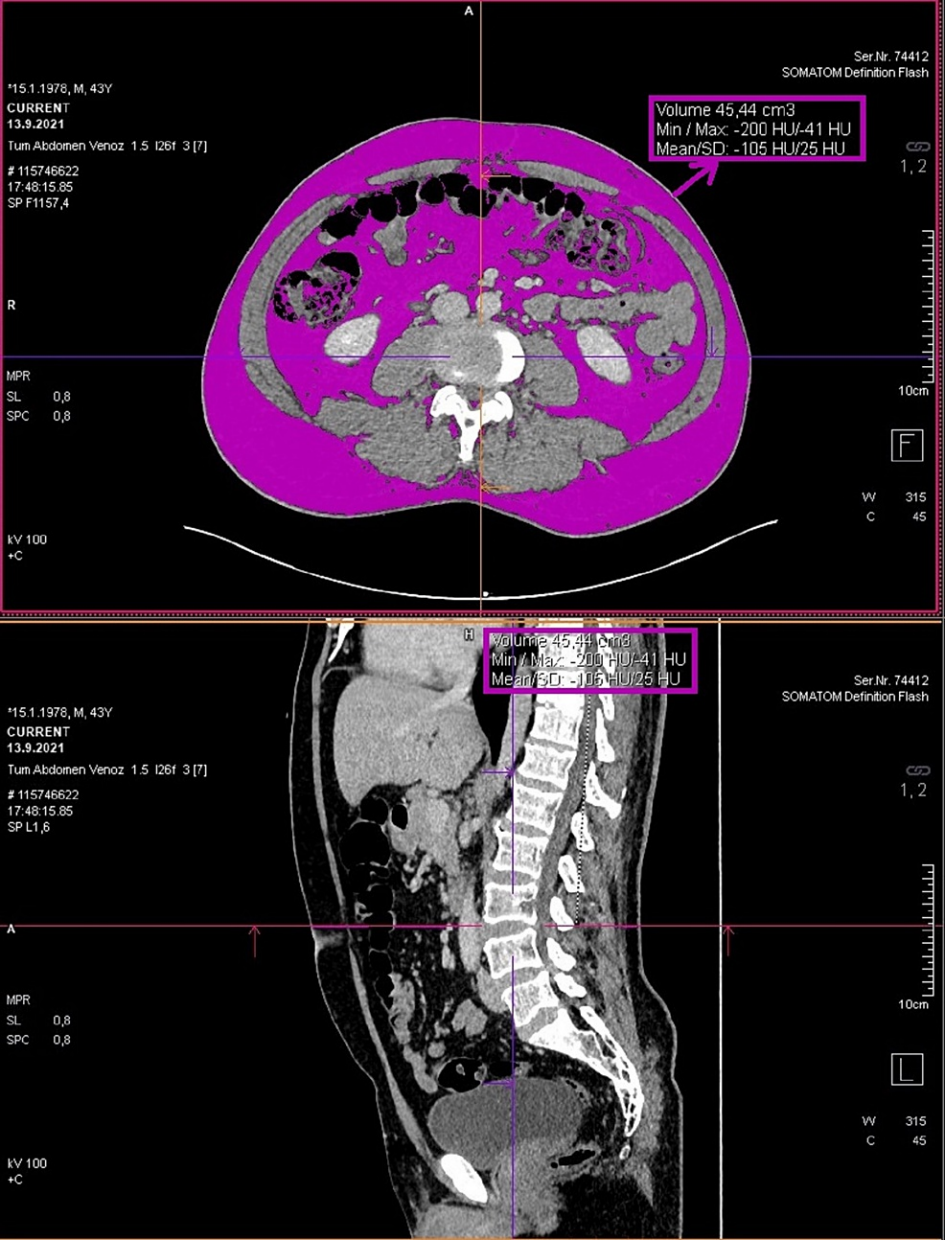
Measurement of total fat volume over fat density by taking the skin line as the border. Calculation of subcutaneous fat tissue volume by subtracting visceral adipose tissue volume from total fat volume.

The density range of -200, -40 HU was selected for the fat density measurement in the cross-section with the "region grooving" application in the angled axial images obtained parallel to the disc plane at this level. First, the fat volume in the whole section was measured (visceral and subcutaneous). Then, only the visceral adipose tissue volume was calculated by drawing borders to exclude subcutaneous adipose tissue (Figure 2). The subcutaneous fat tissue volume was obtained by subtracting the visceral fat tissue volume from the total fat volume (Figure 3).

With the same application, muscle density was selected and paravertebral muscle tissue volume was calculated (bilateral musculus psoas major, musculus quadratus lumborum, musculus iliocostalis, musculus longissimus, musculus multifidus volumes). A Spearman correlation model was used to analyze visceral adiposity, subcutaneous fat, and muscle mass.

In CT images, each intervertebral disc space was evaluated in terms of the presence of osteophytes, loss of disc height, sclerosis in the end plates, and spinal stenosis (spinal canal narrowing under 15 mm AP diameter) to investigate the presence of degeneration. Each level was scored according to the presence of findings, with 1 point for the presence of osteophytes, loss of disc height, sclerosis in the end plates, and spinal stenosis. The total score at all levels (L1-S1) was calculated for each patient.

Statistical analyses were performed using IBM SPSS version 20.0 software (IBM Corp., Armonk, NY). The conformity of the data to normal distribution was assessed using the Shapiro-Wilk test. Normally distributed variables were presented as mean±standard deviation and those not showing normal distribution as median (minimum-maximum) values. Categorical variables were presented as numbers (n) and percentages (%). The Spearman's rank correlation coefficient test was used to determine the correlation between the measured parameters in various vertebral pathologies. Continuous variables were compared using the Mann-Whitney U test. The receiver operating characteristic (ROC) analysis was used to detect the area under the curve (AUC) and define the cutoff values with their sensitivities and specificities of the measurements. An alpha value of p<0.05 was accepted as statistically significant.

## Results

A positive correlation between visceral fat, subcutaneous fat, and total fat was observed. No correlation was detected between any fat volume and total muscle volume (r = 0.450-0.867) (Table 1).

**Table 1:** Correlation between total fat, subcutaneous fat, visceral fat, and total muscle amounts according to Spearman's rank correlation coefficient analysis. *Measurements that are correlated with each other. n: number; SD: standard deviation

Variables	n	Mean (cm³)	SD	1	2	3	4
1. Total fat	146	45.06	1.70	-	0.789*	0.867*	0.115
2. Visceral fat	146	17.91	0.86	0.789*	-	0.450*	0.158
3. Subcutaneous fat	146	26.89	1.20	0.867*	0.450*	-	0.027
4. Total muscle	146	10.13	0.27	0.115	0.158	0.027	-

The associations between the measured parameters (total, visceral, and subcutaneous fat mass, muscle mass) and the presence of loss of height in the vertebral disc, sclerosis, osteophytes, and spinal stenosis are assessed and presented in Tables 2-5. A positive correlation was found between the loss of intervertebral disc height at all lumbar levels and the amount of fat volumes (total, visceral, subcutaneous) (p = 0.001-0.029). Although the amount of muscle volume was not associated with the loss of intervertebral disc height (p = 0.057-0.417).

**Table 2:** The association between the loss of disc height (LDH) and body components. AUC: area under the curve

	Risk factor	AUC (95%)	Cut-off	p-Value	Sensitivity	Specificity
L1-L2 LDH	Total fat	0.704	48.99	0.001	0.322	0.678
	Visceral fat	0.715	20.27	0.001	0.304	0.696
	Subcutaneous fat	0.635	26.1	0.021	0.391	0.609
	Total muscle	0.452	9.45	.417	0.513	0.487
L2-L3 LDH	Total fat	0.697	48.6	0.001	0.667	0.664
	Visceral fat	0.706	19.28	0.001	0.667	0.664
	Subcutaneous fat	0.633	26.1	0.020	0.606	0.611
	Total muscle	0.423	9.26	0.177	0.455	0.442
L3-L4 LDH	Total fat	0.683	47.6	0.001	0.658	0.657
	Visceral fat	0.700	18.65	0.001	0.658	0.648
	Subcutaneous fat	0.619	25.8	0.029	0.632	0.620
	Total muscle	0.396	9.21	0.057	0.421	0.417
L4-L5 LDH	Total fat	0.668	45.76	0.001	0.635	0.638
	Visceral fat	0.668	17.6	0.001	0.615	0.617
	Subcutaneous fat	0.623	25.05	0.014	0.596	0.606
	Total muscle	0.418	9.26	0.100	0.423	0.404
L5-S1 LDH	Total fat	0.693	43.85	0.001	0.652	0.658
	Visceral fat	0.688	16.81	0.001	0.652	0.658
	Subcutaneous fat	0.633	24.5	0.006	0.609	0.618
	Total muscle	0.418	9.45	0.088	0.435	0.434

**Table 3:** The association between the osteophytes and body components.

	Risk factor	AUC (95%)	Cut-off	p-Value	Sensitivity	Specificity
L1-L2 Osteophytes	Total fat	0.644	44.04	0.003	0.576	0.575
	Visceral fat	0.655	17.35	0.002	0.593	0.598
	Subcutaneous fat	0.604	24.5	0.033	0.559	0.563
	Total muscle	0.500	9.55	0.995	0.492	0.494
L2-L3 Osteophytes	Total fat	0.619	44.0400	0.014	0.571	0.429
	Visceral fat	0.635	17.2150	0.005	0.587	0.413
	Subcutaneous fat	0.584	24.5000	0.041	0.556	0.444
	Total muscle	0.479	9.5500	0.671	0.476	0.524
L3-L4 Osteophytes	Total fat	0.597	43.6050	0.044	0.557	0.443
	Visceral fat	0.625	15.5500	0.010	0.570	0.430
	Subcutaneous fat	0.574	24.2500	0.042	0.557	0.443
	Total muscle	0.447	9.5500	0.274	0.468	0.532
L4-L5 Osteophytes	Total fat	0.647	42.7000	0.003	0.596	0.404
	Visceral fat	0.639	15.0500	0.006	0.606	0.394
	Subcutaneous fat	0.629	23.6500	0.010	0.585	0.415
	Total muscle	0.444	9.5500	0.263	0.479	0.521
L5-S1 Osteophytes	Total fat	0.657	42.1000	0.002	0.621	0.379
	Visceral fat	0.651	14.9000	0.003	0.632	0.368
	Subcutaneous fat	0.609	23.6500	0.029	0.579	0.421
	Total muscle	0.446	9.5500	0.279	0.484	0.516

**Table 4:** The association between sclerosis and body components. AUC: area under the curve

	Risk factor	AUC (95%)	Cut-off	p-Value	Sensitivity	Specificity
L1-L2 Sclerosis	Total fat	0.832	63.6150	0.001	0.875	0.125
	Visceral fat	0.702	20.2700	0.001	0.625	0.375
	Subcutaneous fat	0.777	28.5500	0.021	0.625	0.375
	Total muscle	0.242	8.5500	0.417	0.250	0.750
L2-L3 Sclerosis	Total fat	0.603	45.9650	0.300	0.556	0.444
	Visceral fat	0.601	16.8150	0.311	0.556	0.444
	Subcutaneous fat	0.551	25.5000	0.611	0.556	0.444
	Total muscle	0.320	8.7500	0.072	0.333	0.667
L3-L4 Sclerosis	Total fat	0.790	55.1200	0.001	0.667	0.333
	Visceral fat	0.738	18.5950	0.081	0.600	0.400
	Subcutaneous fat	0.785	28.5500	0.001	0.667	0.333
	Total muscle	0.169	8.4750	0.081	0.267	0.733
L4-L5 Sclerosis	Total fat	0.782	48.7200	0.002	0.654	0.346
	Visceral fat	0.738	17.8900	0.015	0.577	0.423
	Subcutaneous fat	0.765	26.8500	0.006	0.615	0.385
	Total muscle	0.169	8.7500	0.081	0.308	0.692
L5-S1 Sclerosis	Total fat	0.792	44.2900	0.001	0.672	0.328
	Visceral fat	0.758	17.2150	0.001	0.625	0.375
	Subcutaneous fat	0.785	24.5000	0.002	0.625	0.375
	Total muscle	0.169	9.4500	0.582	0.469	0.531

**Table 5:** The association between spinal stenosis and body components. AUC: area under the curve

	Risk factor	AUC (95%)	Cut-off	p-Value	Sensitivity	Specificity
L1-L2 spinal stenosis	Total fat	0.782	57.0300	0.055	0.750	0.250
	Visceral fat	0.498	16.1500	0.990	0.500	0.500
	Subcutaneous fat	0.880	34.7000	0.590	0.750	0.250
	Total muscle	0.485	9.4500	0.919	0.500	0.500
L2-L3 spinal stenosis	Total fat	0.657	48.7200	0.234	0.600	0.400
	Visceral fat	0.452	13.8500	0.718	0.400	0.600
	Subcutaneous fat	0.738	33.6500	0.071	0.800	0.200
	Total muscle	0.504	9.5500	0.979	0.400	0.600
L3-L4 spinal stenosis	Total fat	0.670	49.4750	0.107	0.625	0.375
	Visceral fat	0.477	16.1500	0.826	0.500	0.500
	Subcutaneous fat	0.767	13.3000	0.590	0.875	0.125
	Total muscle	0.359	8.9350	0.180	0.375	0.625
L4-L5 spinal stenosis	Total fat	0.631	47.3750	0.080	0.489	0.772
	Visceral fat	0.550	16.1500	0.506	0.402	0.697
	Subcutaneous fat	0.624	13.3000	0.096	0.482	0.767
	Total muscle	0.419	8.9350	0.279	0.288	0.550
L5-S1 spinal stenosis	Total fat	0.626	45.4750	0.183	0.462	0.791
	Visceral fat	0.674	16.1500	0.067	0.513	0.834
	Subcutaneous fat	0.568	13.3000	0.476	0.405	0.731
	Total muscle	0.356	8.9350	0.130	0.240	0.472

As the area under the curve (AUC) values ​​were presented, it was observed that the association between the visceral and total fat volume and degeneration (loss of disc height) scores at all levels was higher than that of subcutaneous fat volume (Table 2). An association was observed between osteophytes at lumbar levels and all the fat volumes (total, visceral, and subcutaneous) (p = 0.002-0.044). Although, the amount of muscle volume was not associated with lumbar osteophytes (p = 0.263-0.995). When the AUC was examined, it was determined that the parameters most associated with the loss of height in the vertebral disc were visceral and total fat masses (Table 3).

An association was found between sclerosis and all the fat volumes at all lumbar levels (p = 0.001-0.021) but again no correlation was observed between the amount of muscle mass and sclerosis presence (p = 0.081-0.582) (Table 4). It was observed that spinal stenosis at the lumbar levels was not associated with the amount of fat (total, visceral, subcutaneous) at any level (p = 0.055-0.990) and also was not associated with the amount of muscle mass (p = 0.130-0.979) (Table 5). In addition, there was a significant difference between the assessed vertebral disorders in terms of fat tissue, but no difference was observed regarding the amount of muscle mass.

## Discussion

The results determined in our study present the association between visceral, subcutaneous, and total fat masses and muscle mass with LVD. Most individuals in the study were overweight and obese (79%), implying a possible change in lumbar disc characteristics due to increased mechanical load, as noted by Iatridis et al. [17]. According to the general opinion, excess weight causes degeneration in the intervertebral disc structure at histological and macroscopic levels, leading to an acceleration of the lumbar degenerative process [3]. In an MRI study conducted by Takatalo et al., it was revealed that there is a relationship between degenerated discs and abdominal obesity [6]. The causal relationship between the height of visceral, subcutaneous, and total fat masses detected in our study and disc degeneration is consistent with the results of the study of Takatalo et al. [6]. Again, Hershkovich et al. reported a relationship between obesity and disc degeneration in terms of low back pain [18]. In addition, vertebral osteophytes and sclerosis were also examined in our study, and the relationship between vertebral bone degeneration and abdominal fat volumes was also revealed.

Although obesity has been shown to be associated with many endocrine and cardiovascular diseases, its relationship with LVD remains unclear in the current literature. The reason is largely associated with the lack of large epidemiological studies with assumptions resulting from an appropriate study design, inadequate statistical analysis, and limited radiographic interpretation of additional spinal findings that may advance to the degenerative process. In a study conducted in the Netherlands in which direct roentgenograms of 2819 individuals were examined, no correlation was found between increased body mass index and decreased intervertebral disc height [19]. Similarly, in a study conducted in England, it was stated that this relationship was weak [20]. Again, in a study conducted in the USA, this relationship was examined in 187 individuals, facet joint degeneration was more common in individuals with increased body mass index, but no relationship was found with the narrowing of the disc space [21]. As can be seen, when investigating the relationship between disc degeneration and vertebral pathologies, the presence of obesity alone seems insufficient which directed us to investigate more related parameters like visceral, subcutaneous, and total abdominal fat volumes.

Previous studies have reported that high BMI is a risk factor for lower back pain. Excessive adipose tissue has been highly blamed for damage to the spinal structures [22]. Structural damage and pathological changes in the vertebral body are the most prominent changes [23]. However, as stated in previous studies, we made these measurements with the thought that the relationship between vertebral bone degeneration and adipose tissue might be more illuminating since BMI has a weak relationship with degeneration. Furthermore, the distribution of the body adiposity may play a more important role in lumbar disc herniation. It has been found that obesity leads to an increase in the synthesis of proinflammatory cytokines produced from adipose tissue. These adipose cytokines also increase c-reactive protein synthesis from hepatocytes in obese individuals [24]. These reasons play a role in the association of obesity with disc degeneration. In our study, lumbar vertebrae degeneration was significantly associated with adipose mass parameters, while none of the muscle mass measurements were related to disc degeneration. Failure to find a relationship between paravertebral muscle volume and vertebral degeneration may mean that the amount of abdominal fat volume may be more effective on vertebral degeneration than the amount of muscle volume, but broader sample size studies are required to advocate this theory.

The fact that vertebral degeneration can also be seen in asymptomatic individuals has led to further investigation of the relationship between disc degeneration and vertebral anatomical differences. Boden et al. performed MRI examination in 67 patients who never had low back pain (LBP), neurogenic claudication, or sciatica, and found that approximately one-third of these patients had significant vertebral pathologies, such as herniated nucleus pulposus, stenosis, degeneration, and bulging [25]. Although this degeneration is observed in asymptomatic individuals, according to Samartzis et al., existing disc degeneration is guiding and predictive for future LBP [26].

Samartizis et al. stated that obesity is a risk factor for the presence, prevalence, and severity of disc degeneration [27]. Takatalo et al. measured body fat using MRI and found similar measurements of abdominal circumference, therefore they suggested clinical use of this measurement to assess abdominal adiposity as a risk factor for disc degeneration [6]. Other studies such as Han et al. reported that an increase in the amount of fat around the abdomen and high BMI values ​​in patients were associated with chronic low back pain and lumbar disc herniation [28]. In another study in the literature indicating the relationship between lumbar fat mass and lumbar intervertebral pathologies, it was reported that subcutaneous fat mass reliably differentiated patients with chronic low back pain and severe Modic changes at the lumbar level from asymptomatic subjects [9]. Baek et al. argued that decreased muscle mass and increased fat mass are associated with the loss of disc height and spondylolisthesis of consecutive vertebrae in the lumbar region [29]. Considering the results of our study, while the relationship between intervertebral disc degeneration and loss of disc height and increased fat mass was consistent with the study of Baek et al., we could not find a relationship between muscle mass and disc pathologies [29].

Our study seems to be quite compatible with the inferences mentioned in these studies, which stated that vertebral disc degeneration is associated with high abdominal adiposity. In our study, we found that the visceral, subcutaneous, and total adipose tissue volumes that we measured in patients who underwent abdominal CT imaging were correlated with each other, but we did not detect any relationship between these adipose tissue measurements and muscle mass. We found that there was a correlation between the loss of vertebral disc space and adipose tissue volumes at all levels. When examined separately, it was observed that the amount of visceral and total adipose tissue was more associated with degeneration than the amount of subcutaneous adipose tissue. Likewise, it is observed that the amount of adipose tissue is associated with vertebral osteophyte and sclerosis formation, and spinal stenosis. We observed that muscle mass was not associated with any of these pathologies. In previous studies, the weak correlation of body mass index alone with vertebral pathologies led us to examine the effect of abdominal fat and muscle amounts. As a result, it was determined in our study that abdominal visceral, subcutaneous, and total fat volumes are associated with pathologies, such as vertebral degeneration, loss of disc height, sclerosis, and osteophyte formation. However, to prove that this relationship is stronger than BMI, it is necessary to examine the correlation between adipose tissue measurements and BMI, which is among the main targets in our future planned studies.

This study aimed to identify the amount of the body composition components like visceral, subcutaneous, and total fat as well as muscle mass as risk factors for loss of disc height (LDH). We believe that our study will make a substantial contribution to the current literature as one of the studies investigating the etiology of vertebral discopathies.

The first and probably the most important limitation of this study is its cross-sectional design. The measurements of this study require further analysis and verification to visualize whether the patients’ fat and muscle composition can predict future lumbar disc pathologies.

## Conclusions

The amount of visceral, subcutaneous, and total adipose tissue in the abdominal region are components associated with vertebral disc degeneration, sclerosis, and osteophyte formation. Abdominal fat mass can be used in clinical decisions as a risk factor for LVD. These factors should be taken into account when assessing the patient's likelihood of developing vertebral disorders.
